# Zinc Oxide Phytonanoparticles' Effects on Yield and Mineral Contents in Fruits of Tomato (*Solanum lycopersicum* L. cv. Cherry) under Field Conditions

**DOI:** 10.1155/2021/5561930

**Published:** 2021-06-10

**Authors:** Federico Antonio Gutiérrez-Miceli, María Ángela Oliva-Llaven, María Celina Luján-Hidalgo, María Concepción Velázquez-Gamboa, Daniel González-Mendoza, Yazmin Sánchez-Roque

**Affiliations:** ^1^Plant Tissue Culture Laboratory, Tecnológico Nacional de México/I.T. de Tuxtla Gutiérrez, Carretera Panamericana Km 1080, Terán, C.P., Tuxtla Gutiérrez 29050, Chiapas, Mexico; ^2^Facultad de Medicina Veterinaria y Zootecnia, Universidad Autónoma de Chiapas, Copainalá, Chiapas, Mexico; ^3^Instituto de Ciencias Agrícolas, Universidad Autónoma de Baja California (ICA-UABC), Carretera a Delta S/N C.P., Ejido, Nuevo León 21705, Baja California, Mexico; ^4^Research Laboratory, Universidad Politécnica de Chiapas, Carretera Tuxtla-Villaflores Km 1 + 500, Las Brisas, C.P., Suchiapa 29150, Chiapas, Mexico

## Abstract

The use of phytonanoparticles in agriculture could decrease the use of fertilizers and therefore decrease soil contamination, due to their size being better assimilated in plants. It is important to mention that the nanofertilizer is slow-releasing and improves plant physiological properties and various nutritional parameters. The influence of soil and foliar applications of phytonanoparticles of ZnO with the *Moringa oleifera* extract under three concentrations (25, 50, and 100 ppm) was evaluated on the cherry tomato crop (*Solanum lycopersicum* L.). Synthesis of the phytonanoparticles was analyzed with ultraviolet-visible spectroscopy (UV-Vis) and infrared transmission spectroscopy with Fourier transform (FT-IR), as well as the analysis with the dynamic light scattering (DLS) technique. The morphometric parameters were evaluated before and after the application of the nanoparticles. The minerals' content of fruits was done 95 days after planting. Results showed that soil application was better at a concentration of 25 ppm of phytonanoparticles since it allowed the greatest number of flowers and fruits on the plant; however, it was demonstrated that when performing a foliar application, the fruit showed the highest concentrations for the elements Mg, Ca, and Na at concentrations of 511, 4589, and 223 mg kg^−1^, respectively.

## 1. Introduction

The application of fertilizers has increased food production considerably; however, the intensive use of these significantly threatens environmental health and ecosystem functions. Most of the fertilizers are not available to plants due to run-off and cause pollution. Fertilizers coated in nanomaterials can solve this problem [1]. The bionanoparticles' synthesis is an ecologically sound and sustainable alternative to the conventional methods, in as much as bionanoparticles can use easily available bio-resource from the leafs extract of a plant, such as the case of phytonanoparticles (NPs). The use of practically nontoxic natural extracts allows the green synthesis of NPs and being used in sensitive areas, such as agriculture [2]. Nanocatalysis represents nowadays an innovative approach to obtain better properties due to stable activity and good selectivity [3].

In addition, zinc oxide is currently used as an antimicrobial agent in micro- and nanoscale formulations; these nanoparticles could be used in agriculture as a plant protector against pathogens and in fertilizer products as micronutrients [4]. This formulation could bring benefits for plants and/or edible crops [5] of economic importance such as the tomato (*Solanum lycopersicum* L.). Specifically, the cherry tomato is characterized by small fruits with different sizes, colors, and flavors. It is currently in high demand in restaurants and bars and in the manufacture of various dishes as gourmet snacks [5].

Mineral's uptake in adequate levels plays important role in growth, yield, and quality attributes of tomato and also contains antioxidant compounds important for human health; however, in recent years, a deficiency of mineral elements of up to 80–90% has been observed in fruits. This can be attributed to conventional agricultural techniques [4].

Therefore, given this deficiency present in tomato plants and fruits, the present research aimed to evaluate the effect of ZnO phytonanoparticles with aqueous extract of *Moringa oleifera* on the accumulation of mineral elements in tomato plant fruits (*Solanum lycopersicum* L.) through an edaphic and foliar application.

## 2. Materials and Methods

### 2.1. Experimental Area

The experiment was carried out at the Ranch “El capricho divino” located in the municipality of Suchiapa, Chiapas, Mexico, located at 16° 37′ 30″ north latitude and 93° 6′ 0″ west longitude, at an altitude of 500 m above sea level, with warm subhumid climate and periodic rains; the average annual temperature in the municipal seat is 24.4°C, with a rainfall of 956 mm per year.

### 2.2. Green Synthesis of Nanoparticles

The methodology described by [6] with some modifications was used, so 15 mL of the aqueous extract of *Moringa oleifera* was added to 1.64 g of zinc (ZnSO_4_); the reaction mixture was kept under stirring for 6 h and, subsequently, 2 m of NaOH was added to the solution and placed in an incubator at 60°C with constant shaking for 12 h. The colloidal solution was centrifuged at 4500 rpm for 20 min, and the precipitate was subjected to two consecutive washes with 96% ethanol and distilled water. Finally, the precipitate was dried in an oven at 50°C and a fine powder was obtained with the help of a mortar.

### 2.3. Physicochemical Characterization of Green Nanoparticles

The identification of the production of the ZnO phytonanoparticles was first evaluated by the color change of the aqueous solution of *M. oleífera* by Abdelmoteleb et al. [7].

For the characterization of ZnO phytonanoparticles, ultraviolet-visible spectroscopy (UV-Vis) and infrared transmission spectroscopy with Fourier transform (FT-IR) were first carried out. This was verified by performing a spectral scan of the colloidal solution in a range of 300–700 nm in a UV-Vis spectrophotometer (Beckman Coulter, USA), calibrated with a ZnSO_4_ solution (0.34 m) [7]. Likewise, the presence of the bands corresponding to the zinc metallic bonds in the phytonanoparticles was identified with an infrared spectroscope (NICOLET, Wisconsin, USA).

The size of the phytonanoparticles and the zeta potential were analyzed in solution using a nanotrac wave instrument (Microtrac, Montgomeryville PA, USA) according to Mendez-Trujillo et al. [8]. Measurements were made using the dynamic light scattering (DLS) technique in a range of 0.1–1000 *μ*m at 25°C, with a laser wavelength of 780 nm and a scattering angle of 90 Åã. Data obtained from DLS was analyzed using Microtrac FLEX operating software [9–11].

### 2.4. Experiment Establishment

The crop used was *Solanum lycopersicum* L. cv cherr. Five rows were planted with 100 seedlings each, the cultivation had a drip irrigation system and tensioned ropes in order to support the stem in its development in a ½ ha antiaphid house. During their growth, phytonanoparticles of zinc oxide were applied in different concentrations (0, 25, 50, and 100 ppm) applying edaphically under the irrigation lines of the crop and in a foliar way through the use of a sprinkler; the first row was omitted or the first seedling of each row to decrease the ambient noise factor, and the treatments were applied once a week for 3 weeks.

Morphometric parameters in relation to the growth of the crop, such as plant height, number of fruits, and number of flowers, were evaluated before and after the application of the nanoparticles every 10 days for 60 days [5, 12].

### 2.5. Fruit Characterization

The fruits were characterized followed by the first harvest in a time of 95 days after planting. The method used was an adaptation of method 3052 described by U.S. EPA (Environmental Protection Agency), where 300 mg of the dried and pulverized plant material was subjected to acid digestion with 9 mL of nitric acid, 2 mL of 30% hydrogen peroxide, and 1 mL of hydrochloric acid at 180°C until total digestion of the plant material [13]. After the digestion, the samples were filtered by Whatman No. 40 paper and graduated to 25 mL. The minerals quantification was performed using plasma emission spectrometry (ICP-OES) (Perkin Elmer, Kyoto, Japan).

The total soluble solids in the fruits were determined using a digital refractometer. Fruits were sampled in triplicate of each treatment (each sample one gram) [14, 15].

## 3. Results and Discussion

### 3.1. Physicochemical Characterization of Green Nanoparticles

**Figure 1:**
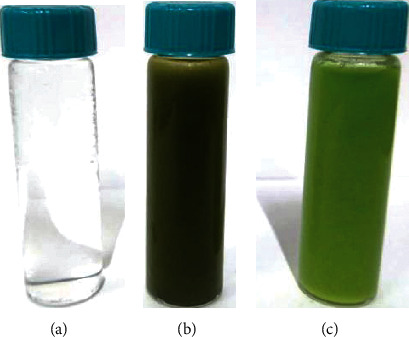
Colloidal solution of phytonanoparticles from the aqueous extract of *Moringa oleifera*: (a) ZnSO_4_ solution, (b) *Moringa oleifera* extract, and (c) ZnO phytonanoparticles after 12 hours of incubation at 60°C.

During the green synthesis of zinc oxide nanoparticles using the aqueous extract of *Moringa oleifera*, a change in coloration was observed in the extract on contact with the ZnSO_4_ solution, due to the reduction of Zn^+2^ to Zn^0^ (Figure 1).

**Figure 2:**
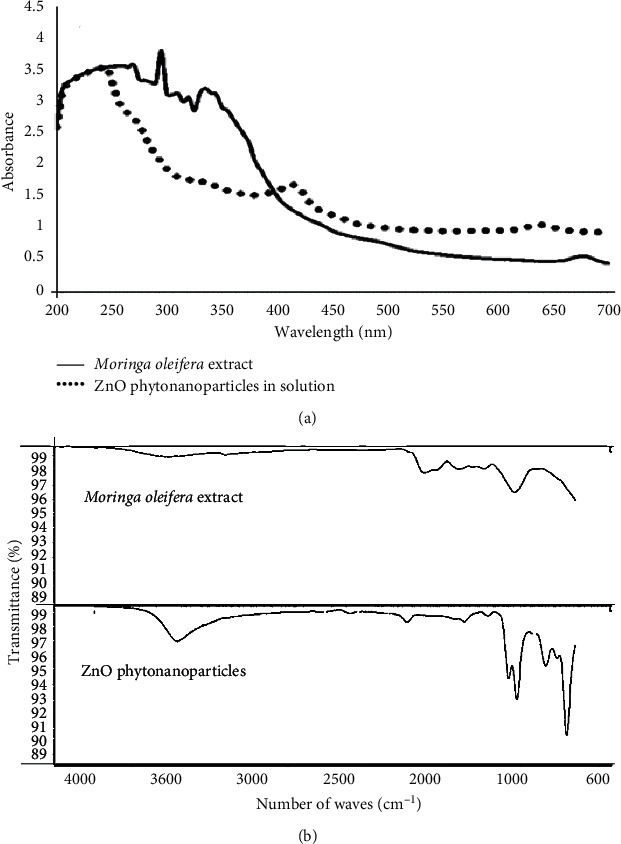
Characterization of ZnO phytonanoparticles with the aqueous extract of *Moringa oleifera*: (a) UV-Vis absorption spectrum and (b) FT-IR absorption spectrum.

The UV-Vis absorption spectrum of the colloidal solution is shown in Figure 2(a), the solid line corresponds to the plant extract of *Moringa oleifera*, and absorption peaks were observed approximately between 300 and 350 nm. On the contrary, the dotted line corresponds to the spectrum of absorption of the phytonanoparticle solution, highlighting the absorption peak at approximately 400 nm. Also, the IR adsorption spectrum of the dry ZnO phytonanoparticles is presented in Figure 2(b), adsorption bands were identified at 3390 and 2825 cm^−1^, and these bands are characteristic of the vibrations by stretching of the O-H and C-H bonds, respectively. The band at 1830 cm^−1^ can be attributed to the asymmetric and symmetrical stretching of zinc carboxylates [16], while the band at 871 cm^−1^ is due to the stretching vibrations of the O-Zn bonds [17].

**Figure 3:**
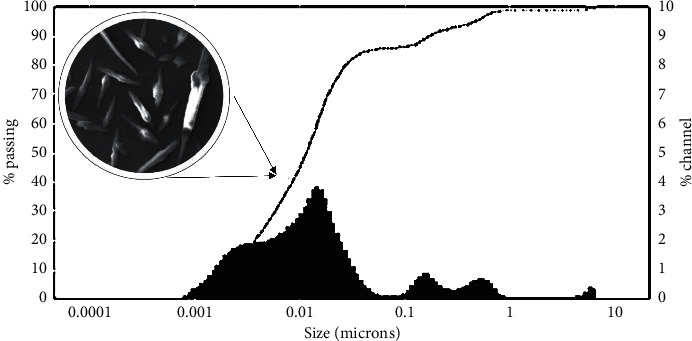
Particle size distribution of ZnO NPs-*M. oleifera* using dynamic light scattering (DLS) measurements.

The DLS results for particle size in the solution of ZnO phytonanoparticles are presented in Figure 3. The results showed that ZnO NPs from *M. oleifera* form agglomerates with an average hydrodynamic size of 13 um and zeta potential value of 22.56 mV, when dispersed in water.

The synthesis of ZnO phytonanoparticles from *M. oleifera* was confirmed by the change of color from dark green to light green color suggesting that ZnSO_4_ dissociates from Zn^+2^ to Zn^0^ by reduction action of phytochemicals present in *M. oleifera* phytonanoparticles. This behavior was similar to that reported by Ruiz-Romero et al. [18], who attributed the change in hue in the solution to the formation of zinc oxide nanoparticles.

During the UV-Vis absorption analysis of the aqueous extract of *M. oleifera* extract, peaks between 300 and 350 nm were identified (Figure 2(a)). These peaks correspond to the light absorption of the *π* bonds of the benzene rings of the phenolic compounds present in the extract [19], while the absorption spectrum of phytonanoparticles in the solution presented a band of maximum absorption at 400 nm (Figure 2(b)). These irregularities are associated with chemical modifications with the reduction of metal ions and the oxidation of antioxidant compounds [20].

The zeta potential is an important characteristic that must be known about phytonanoparticles, since this characteristic influences other of equal importance, such as saturation solubility, dissolution rate, physical stability, and even biological performance [21]. A minimum of zeta potential (+25 mV or −25 mV) demonstrates stable nanostructures [22]. As the zeta potential approaches “0” or the zero charge point, the attraction between the nanomaterials exceeds the repulsive forces between the structures resulting in agglomeration. The zeta potential values for the ZnO phytonanoparticles obtained in the present study indicate long-term stability of the colloids; this could be attributed to the presence of bioactive components in the aqueous extract of *M. oleifera* which covers the nanoparticles, stabilizing them.

### 3.2. Evaluation of Morphometric Parameters of Tomato Plants

**Figure 4:**
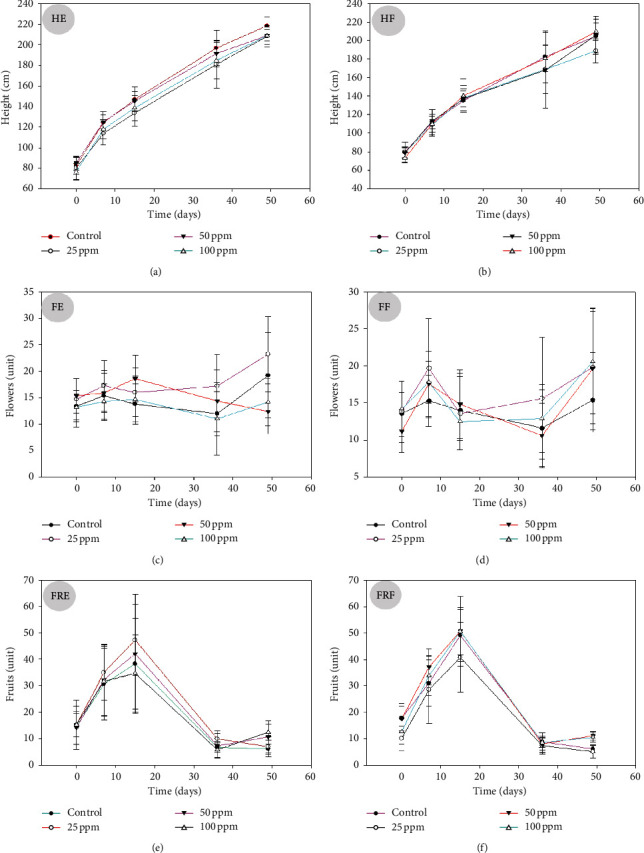
Evaluation of morphometric variables of the effect of ZnO phytonanoparticles with the *Moringa oleifera* extract on the tomato plants (*Solanum lycopersicum*). Mean of three repetitions with standard error. HE: height/edaphic; HF: height/foliar; FE: flowering/edaphic; FF: flowering/foliar; FRE: fruiting/edaphic; FRF: fruiting/foliar.

The height of the tomato plants was evaluated as a morphometric variable, and greater growth was observed in terms of the application of the phytonanoparticles on the root at a concentration of 50 ppm; however, no significant statistical differences are seen with the control treatment (Figure 4 HE-HF). Since zinc nanoparticles have shown to induce free radical formation, resulting in increased malondialdehyde and lower levels of reduced glutathione and reduced chlorophyll contents [8], this significantly impacts photosynthesis processes limiting development.

Likewise, the influence of phytonanoparticles on flower production was evaluated; specifically, a significant increase is observed in the treatment that applies 25 ppm of phytonanoparticles through the root, producing a total of 23 flowers and showing significant statistical differences with the rest of the treatments. However, it is important to mention that according to the edaphic application of phytanoparticles, a decrease in flowering was observed as the concentration of applied phytonanoparticles increased (Figure 4, FE-FF) (Table 1).

So too, soil application of phytonanoparticles resulted in the highest fruit number per plant, since it allowed the production of 48 fruits at 15 days at a concentration of 100 ppm, demonstrating significant statistical differences with the rest of the treatments and the control (Figure 4, FRE-FRF) (Table 1). As was demonstrated by García-López et al. [11] who evaluated ZnO nanoparticles, they mentioned that application of ZnO phytonanoparticles (NPs) could be employed in habanero pepper production to improve yield and quality, but it is still unknown nutraceutical properties of fruits.

However, Santhoshkumar et al. [10] evaluated the use of nanoparticles of *Moringa oleifera*; they identified that the green synthesis of phytonanoparticles significantly improves the flowering and fruiting of crops, as observed in this research paper (Figure 4). The use of plant materials for green synthesis contains certain bioactive compounds like flavonoids, phenols, citric acid, ascorbic acid, polyphenolic, terpenes, alkaloids, and reductase which act as reducing agents. Plant-mediated synthesis of nanoparticles is a very promising area of nanotechnology because the plant itself acts as both a reducing and capping agent [4, 19].

The promoter effect could be related to the zinc role as a precursor in the synthesis of auxins that promote cell division, as well as its influence on the reactivity of indoleacetic acid, which functions as a hormonal phytostimulant [8, 13, 17, 18]. Furthermore, it is possible that ZnO nanoparticles could be involved in a greater production of the phytohormones cytokine and gibberellin, apart from inducing the activity of antioxidant enzymes [23].

Likewise, Santos-Espinoza et al. [24] indicated that the mechanisms by which peanut plants respond positively to the application of nanoparticles were an increase in the activity of phenylalanine ammonia-lyase and antioxidant enzyme. They revealed a significant decrease in indole-3 acetic acid and induced the synthesis of gibberellins; the improvement in the quality of peanut fruits was demonstrated.

Abdelmoteleb et al. [7], mentioned that nanoparticles have great benefits in agriculture, since the phytocomposites nanoencapsulated help in slow and sustained release of nutrients and agrochemicals resulting in precise dosage to the plants because of holding the material more strongly from the plant due to higher surface tension of nanoparticles than conventional surfaces.

### 3.3. Fruit Characterization

**Figure 5:**
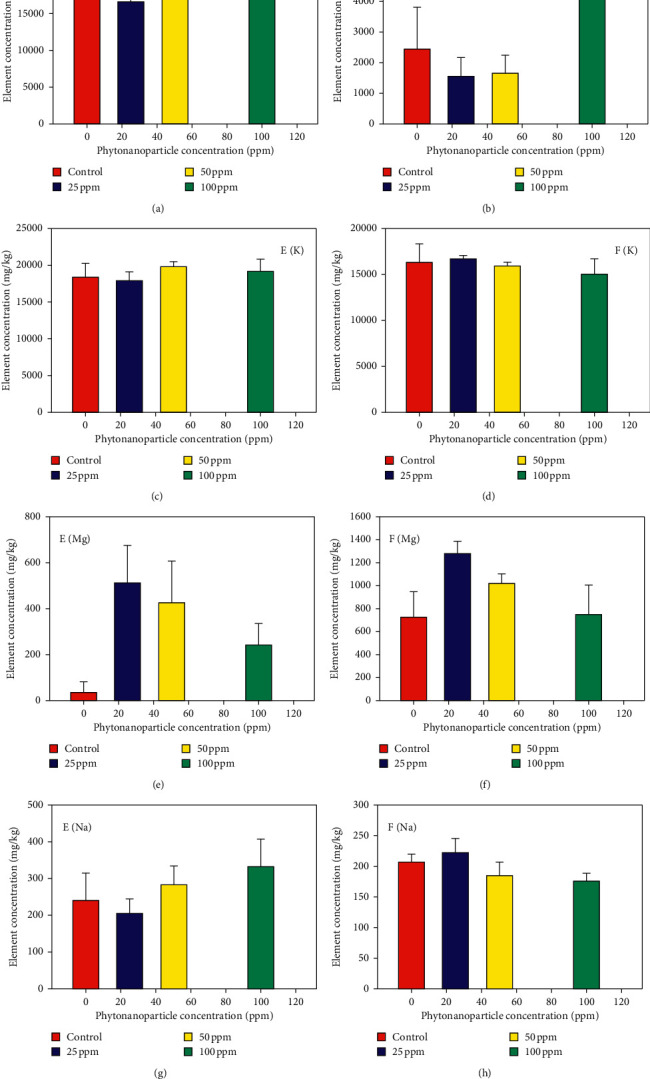
Evaluation of the accumulation of mineral elements in the fruits of tomato plants (*Solanum lycopersicum*) in relation to the effect of ZnO phytonanoparticles with the *Moringa oleifer*a extract. Mean of three repetitions with (±) standard error. Application: E: edaphic; F: foliar.

After the harvest, the effect of the phytonanoparticles applied in the root and on the leaves at different concentrations was evaluated on the bioaccumulation of chemical elements in the fruits. Soil application at 25 ppm level showed a statistically significant difference in the accumulation of magnesium compared to the other treatments and the control, demonstrating the 10-fold increase in the bioaccumulation of this element (Figure 5, EMg). Regarding sodium, it was observed that, at an application of 100 ppm, there was an increase of 42.22% in the concentration of this element in the fruit compared to the control treatment (Figure 5, ENa). On the contrary, it also increased calcium concentrations by a 5.71% when 50 ppm of phytonanoparticles are applied at the root level compared to the control (Figure 5, ECa), however, when evaluating the potassium element, no significant differences were demonstrated compared to the control treatment (Figure 5, EK) (Table 1).

Regarding the foliar application of different concentrations of phytonanoparticles, an increase of 85.71% of the magnesium element was observed in the fruit when 25 ppm was applied, as well as an increase in calcium and sodium of 94% and 11.94%, when applying 100 and 25 ppm, respectively (Figure 5, FCa-FNa), showing significant statistical differences with respect to the control treatment. However, there are no significant differences in the bioaccumulation of the potassium element with respect to the control (Figure 5, FK) (Table 1).

It was shown that magnesium is the element with the highest accumulation in both forms of application (Figure 5); however, a higher concentration of this element was demonstrated when the application is at the foliar level. Li et al. [25] reported that nonglandular trichomes (NGTs) were particularly important for foliar Zn absorption and moved across the cuticle before accumulating in the walls of the epidermal cells. Once absorbed, the Zn accumulated in the walls of the epidermal and the vascular cells and trichome bases of both leaf sides, with the bundle sheath extensions. The said saturation affects photosynthetic efficiency and therefore impacts flower formation and fruiting.

Also, Guo et al. [26] stated that the bioaccumulation of Mg participates in some signaling pathways for hormone production that participate in the carbon fixation, perspiration, accumulation starch, sugar metabolism, and ion up taking, important biological processes to the growth of plants [27].

According to the aforementioned, the significant increase in magnesium identified in this research work is a very important finding, since magnesium is a critical mineral in the human body and is involved in ∼80% of known metabolic functions. It is currently estimated that 60% of adults do not achieve the average dietary intake (ADI) and 45% are magnesium deficient, a condition associated with disease states like hypertension, diabetes, and neurological disorders, to name a few [28, 29].

On the contrary, in the present research work, the highest bioaccumulated concentrations of the elements calcium and sodium were observed, when phytonanoparticles were applied in a foliar way (Figure 5). Pérez-Labrada et al. [30] discovered that foliar application nanoparticles establish energetic interactions, demonstrating greater accumulation of cationic mineral elements such as Na^+^ and Ca^+^.

In relation to calcium, Tien et al. [31] demonstrated that forms of calcium regulate physiological processes such as the secretions of airway epithelia and exocrine glands, the contraction of smooth muscles, and the excitability of neurons; therefore, it is important to have natural sources that provide calcium.

Thus, the increase in calcium in the fruit generates great benefits at a commercial level, since it provides the fruit with more resistance during postharvest handling, even more important if we speak of fruits as perishable as the tomato [32]. As previously mentioned, Sinha et al. [33] identified that calcium (2.0%) enhanced the storage life of plum fruits by reducing physiological loss in weight (PLW), total sugars and pectin methylesterase (PME), and cellulase activities; delaying the development of fruit color and anthocyanin; and retaining sensory quality, firmness, and total sugars in plum fruits. Also, Hyodo et al. [34] identified that the Ca-binding pectin and hairy pectin in skin cell layers are important for intercellular and tissue-tissue adhesion. Maintenance of the globular form and softening of tomato fruit may be regulated by the arrangement of pectin structures in each tissue; therefore, by increasing the calcium concentrations in the fruit, it improves the turgidity and texture of the fruit [35, 36].

On the contrary, the importance of sodium in fruits is also evident, since Stamler et al. [37] mentioned that controlled and low consumption of sodium, from natural sources, such as fruits, generates an osmotic balance of blood pressure [38].

**Figure 6:**
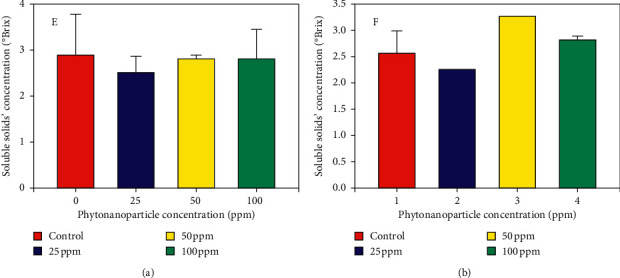
Quantification of total soluble solids in fruits of tomato plants (*Solanum lycopersicum*) in relation to the effect of ZnO phytonanoparticles with the *Moringa oleifera* extract. Mean of three repetitions with (±) standard error. Application: E: edaphic; F: foliar.

Moreover, when evaluating the total soluble solids, an increase of 26.92% was observed with respect to the control, when 50 ppm of phytonanoparticles was applied in a foliar way (Figure 6) (Table 1). It is important because it indicates the amount of sugar (sucrose) present in the fruit, as well as the freshness and the state of maturity of the same; this variable determines the decision-making for the direction of the fruit before a chain of agroindustrial transformation [39].

In this sense, in the present research work, it has been shown that the application of phytonanoparticles benefits the accumulation of elements such as Mg, Na, and Ca in the fruit, indicating that the stomata were an important pathway for the foliar absorption of ZnO in the plant, due to the retention and slow release of phytonanoparticles [27]; however, with an edaphic application, the direction of the compounds through the root system is guaranteed. This highlights that the slow-releasing nanofertilizer improves plant physiological properties and some nutritional parameters [29].

In this sense, fruits and vegetables (FVs) are recognized as healthy constituents of diet and a sustainable solution to the existing twin burden of micronutrient deficiencies [27]. In general, FVs are nutrient dense foods low in energy, containing varying amounts of vitamins and minerals including sodium, iron, zinc, potassium, calcium, magnesium, and fiber [27]. These are abundantly rich in phytochemicals that function as antioxidants, antiatherosclerotic, and anti-inflammatory agents. Some studies have correlated the health benefits of FVs in relation to cardiovascular disease, obesity, and diabetes [34].

According to the aforementioned, it is important to state that the phytonanoparticles increase the concentration of micronutrients in the fruits [9, 40]. Likewise, all benefits of phytonanoparticles are attributed to the antioxidant activity (AOA) of extracts from plant leaves because of impacts on the kinetics of photosynthesis, the size of the formed nanoparticles, and the stability of their nanosuspensions, due to the fact that the leaf extracts contain a wide range of biomolecules and metabolites, such as terpenoids, vitamins, polysaccharides, proteins, amino acids, alkaloids, (poly)phenolic compounds, aromatic amines, tannins, saponins, ketones, aldehydes, flavonoids, organic acids, enzymes, which act as reducing agents and stabilizer [19, 27, 41].

## 4. Conclusion

In conclusion, it was shown that application of phytonanoparticles in plants may help to optimize fertilization strategies, for maintaining important physiological processes such as net of micro- and macronutrients assimilation that is required for optimal plant growth, yield, and fruits quality by what is known to regulate the mechanisms of homeostasis in higher plants at the level of the cellular function. On the contrary, the phytonanoparticles induced the increase of magnesium, calcium, and sodium in fruits, considering that these minerals play an important role in regulating the biological functions of the consumer.

## Figures and Tables

**Table 1:** Percentage analysis of the increase or decrease of the morphometric variables, accumulation of elements, and total soluble solids, depending on the effect of phytonanoparticles on tomato plants and fruits (*Solanum lycopersicum*).

Morphometric variables' evaluation (%)							Total soluble solids' evaluation (%)	
Concentration (ppm)	Height		Flowering		Fruiting			
	E	F	E	F	E	F	E	F
25	−9.09^*∗*^	−10	+51.17	+38.46	0	0	−13.79	−11.53
50	0	0	−29.41	+38.46	+100	+60	−6.89	+26.92
100	−9.09	0	−17.64	+43.84	+200	+60	−6.89	+3.84

Analysis of accumulated elements in the fruit (%)								
Concentration (ppm)	Ca		Mg		Na		K	
	E	F	E	F	E	F	E	F

25	−2.85	−40	+1000	+85.71	−11.11	+11.94	−2.77	+0.05
50	+5.71	−32	+750	+149.25	+20	−10.44	+11.11	−5.88
100	−5.71	+94	+350	0	+42.22	−12.93	+5.55	−17.64

^*∗*^Mean of three repetitions. Application: E: edaphic; F: foliar; () gray color refers to an increase; (0) zero result means the value remains the same as the control; (100) the result hundred means that the value represents twice the result obtained in the control.

## Data Availability

The data used to support the findings of this study are included within the article.
